# Maximum Relevance Minimum Redundancy Dropout with Informative Kernel Determinantal Point Process

**DOI:** 10.3390/s21051846

**Published:** 2021-03-06

**Authors:** Mohsen Saffari, Mahdi Khodayar, Mohammad Saeed Ebrahimi Saadabadi, Ana F. Sequeira, Jaime S. Cardoso

**Affiliations:** 1 INESC TEC and Faculty of Engineering, University of Porto, 4200-465 Porto, Portugal; jaime.cardoso@inesctec.pt; 2 Department of Computer Science, University of Tulsa, Tulsa, OK 74104, USA; mahdi-khodayar@utulsa.edu; 3 Faculty of Electrical Engineering, K. N. Toosi University of Technology, Tehran 16315-1355, Iran; msedebrahimi@email.kntu.ac.ir; 4 INESC TEC, 4200-465 Porto, Portugal; ana.f.sequeira@inesctec.pt

**Keywords:** deep learning, regularization methods, dropout, determinantal point process, information theory, image classification

## Abstract

In recent years, deep neural networks have shown significant progress in computer vision due to their large generalization capacity; however, the overfitting problem ubiquitously threatens the learning process of these highly nonlinear architectures. Dropout is a recent solution to mitigate overfitting that has witnessed significant success in various classification applications. Recently, many efforts have been made to improve the Standard dropout using an unsupervised merit-based semantic selection of neurons in the latent space. However, these studies do not consider the task-relevant information quality and quantity and the diversity of the latent kernels. To solve the challenge of dropping less informative neurons in deep learning, we propose an efficient end-to-end dropout algorithm that selects the most informative neurons with the highest correlation with the target output considering the sparsity in its selection procedure. First, to promote activation diversity, we devise an approach to select the most diverse set of neurons by making use of determinantal point process (DPP) sampling. Furthermore, to incorporate task specificity into deep latent features, a mutual information (MI)-based merit function is developed. Leveraging the proposed MI with DPP sampling, we introduce the novel DPPMI dropout that adaptively adjusts the retention rate of neurons based on their contribution to the neural network task. Empirical studies on real-world classification benchmarks including, MNIST, SVHN, CIFAR10, CIFAR100, demonstrate the superiority of our proposed method over recent state-of-the-art dropout algorithms in the literature.

## 1. Introduction

Recently, in a wide range of machine learning (ML) studies, neural networks (NNs) play a decisive role as powerful statistical pattern-recognition models inspired by the structure of human brain. During recent decades, a wide variety of NNs have been proposed for computer vision applications. Specifically, the emergence of deep neural architectures has opened new research gates for the sake of achieving the utmost goal of ML, i.e., providing large generalization capacities and avoiding overfitting on the training datasets. Shallow and Deep NNs are widely employed for many aspects of contemporary applications such as fingerprint presentation attack detection [1], sequential modelling of multi-scale energy time series [2] and mobile robot motion control [3] etc. due to large computational power, handling uncertainty factors, and efficient implementation.

Deep NNs have a large parameter space corresponding to a vast number of their tunable variables (e.g., memory vector of long short-term units [4] and filtering variables in convolutional NNs). Hence, these models are prone to overfitting due to unnecessarily large decision boundary nonlinearity. This issue is considered a crucial challenge for deep learning algorithms during the last decade. The existing literature presents several regularization methodologies to overcome this issue. There are generally two classes of approaches for regularization: (1) Starting from the objective function and redesigning this function in a way to avoid large changes during parameter updates, e.g., L2 weight decay [5], decorrelating representation [6], correlational neural networks [7], etc.; (2) Implementing the corresponding regularization strategy via mathematical analysis of the coadaptation/distribution between neural units and/or weights. e.g., batch normalization (BN) [8] gradient augmentation [9] and dropout [10].

Among regularization techniques, dropout has shown more applications due to thinner and sparser sub-network resulting from a probabilistic removal of a subset of neurons from the original network. In contrast to recent applications of the dropout technique that discard the extracted kernels in an identical and independent manner [11,12], this paper defines a novel value metric by proposing semantic merit functions (MFs) to remove from the neural network neurons with low contributions. To highlight the performance of each latent neuron, we present algorithms to drop out kernels irrelevant to the underlying task that lie in the dense layers of the deep structure. First, a semantic layer-wise strategy is devised that works based on the mutual information (MI) within kernels and NN target vectors. This method not only mathematically determines output-relevant hidden units to improve the quantity of information, but also determines the subset of hidden units that share less information between each other which promotes the quality of information. Furthermore, we study the integration of the determinantal point process (DPP) [13] to extract the most diverse information in the underlying neural network’s latent space. In addition, we develop a new variation of the presented algorithm to leverage the information obtained from our MI-based technique into the DPP dropout. In this context, we design the dynamic dropout strategy, i.e., DPPMI dropout, which computes diverse and relevant potential representations for a specific ML task using deep structures. We evaluate our work on different, widely used benchmarks in the area of image classification.

Our main contributions are listed as follows:

1. A novel dropout method, mutual information-based dropout i.e., MI dropout, is presented to optimize the number of latent units in a deep neural architecture using mutual information within the units as well as the task-specific target vector.
2. We develop a new variation of our work using the Determinantal Point Process to extract the most diverse data representations that lie in the latent space of the underlying deep neural network. Furthermore, the integration of MI and DPP is studied which results in extracting high-quality and informative latent representations.
3. An experimental study in image classification tasks, such as digit recognition and small images multi-target detection, demonstrates the superiority of the proposed approaches over state-of-art dropout techniques.

This manuscript is organized as the following: Section 2 presents the related works in the area of dropout regularization. Section 3 presents the proposed algorithm using MI and its variations that leverages DPP. To justify the merit of this research, Section 4 evaluate the presented algorithms on image classification tasks. Finally, Section 5 discusses the conclusions and future works.

## 2. Related Works

Recently, dropout has shown a better efficiency compared to other regularization methods. This category of regularizers temporarily removes task-irrelevant units from the NN architecture, along with all their incoming and outgoing connections [10]. The Standard dropout removes each computational latent unit using a fixed removal probability *p* independent of the rest of latent units. In recent studies, a variety of methods such as Standout [14], Guided dropout [15], Adversarial dropout [16], Automatic dropout [17], and Targeted dropout [18] etc. are proposed to achieve a more semantic dropout mechanism. Generally, dropout methods randomly modify latent unit parameters during the training procedure. An early standard dropout variation is the DropConnect [19] that sets a subset of weights and biases to zero rather than working with the neurons’ outputs. Also, Goodfellow et al. proposed maxout [20] to facilitate dropout optimization, which leads to better classification accuracy. Another adaptive generalization of standard dropout is the evolutionary dropout [21], which computes the dropout sampling probabilities using second-order statistics of neural activations in mini-batches of samples.

Recently, Dodballapur et al. proposed Automatic dropout [17], a simple but effective dropout approach. Automatic dropout is quite similar to standard dropout, and it determines the *p* parameter based on the clusters of activation functions in a layer. Controlled dropout [22] is a more memory efficient and faster version of standard dropout that the authors have suggested to gather and relocate non-zero weights in a new memory. Guided dropout [15] and Concrete dropout [23] are two efficient approaches that seek to find the *p* parameter by minimizing a defined objective function. By making use of the MI concept, Chen et al. [24], proposed DropMI method that selects most relevant neurons to the target vector. In their work, using a fixed threshold value they generate a binary mask to remove less important features from NN.

Although the classic approaches generally focus on neuron removal in dense layers, several studies aim to provide sparse CNN structures. In this line of research, Cutout [25] is presented as a generalization of classic dropout for Convolutional NNs (CNNs). Unlike previous works that apply dropout in the feature extraction level, Cutout randomly masks out square regions on the input samples. A similar method is proposed by Ghiasi et al. [26] to simultaneously drop the contiguous regions of a collection of feature maps. Also, the Stochastic Depth (SD) [27] and Swapout [28] are two training procedures designed for very deep CNNs [29]. SD randomly drops a subset of layers on the training phase while Swapout combines dropout with SD to obtain each output independently by reporting the sum of a randomly selected subset of current and all previous layers’ outputs for that unit.

Recently, dropout has been considered for model compression. Gomez et al. proposed Targeted dropout [18] in which, first, a magnitude-based strategy determined the least relevant units, then dropout is applied to this subset of units. Salehnejad suggests exploiting Ising energy for the determination of irrelevant units [30]. Based on the information bottleneck principle, Achille proposed Information dropout [31], in which a more disentangled representations is computed by injecting multiplicative noise in the activation maps. Moreover, Wang et al. proposed Fast dropout [32] that interprets dropout methods from the Bayesian perspective and reaches the same validation performance with a smaller computation burden. Also, in [33], the β-dropout seeks to unify discrete and continuous dropouts. The authors indicated that adjusting the shape parameter β, β-dropout can yield Bernoulli dropout, Uniform dropout, and approximate Gaussian dropout. In addition, Zoneout [34] is a generalization of dropout for Recurrent Neural Networks (RNNs). In contrast to the classic perspective, which sets units’ activations to zero, Zoneout maintains a random selection nature by randomly swapping units’ activations in the temporal domain, and it merely considers stand-alone latent feature maps. Motivated by these drawbacks, this work proposes new dropout strategies that retain the latent kernels in the model by evaluating of kernels’ performance in NN’s training phase. Moreover, in contrast to [24] which only rates the neurons based on their relevance to the target vector, the proposed approaches not only consider the extracted information among kernels in the latent space but also determine the importance of each kernel with respect to the underlying task. Every unit in the model conveys some information between input and output layers. If we could measure the value of information and find out the content of the information for every unit, we could select the units more wisely, preserve the more informative units, and eliminate specific unimportant units during the dropout. To the best of our knowledge, this is the first work that rigorously takes into account both the quality and quantity of information, leveraging the information theory and determinantal point process tools.

## 3. Method

Let us define a multilayer neural network with *L* hidden computational layers with indices $l\in \left\{1,2,\cdots ,L\right\}$. $W^{l}$ and $b^{l}$ are the weight matrix and bias vector of the *l*th layer with input vector $I^{l}$ and output vector $O^{l}$, respectively. The feed-forward propagation step of this NN is written as:

(1) $$I_{i}^{l+1}=W_{i}^{l+1}O_{i}^{l}+b_{i}^{l+1}$$

(2) $$O_{i}^{l+1}=f_{i}\left(I_{i}^{l+1}\right)$$

where f(•) is the activation function for the *i*th hidden unit.

In the Standard dropout method, each unit is retained with a fixed probability *p* independently of other units, where *p* is chosen using a validation set or can simply be set to 0.5. The dropout method considers a binary mask $M_{i}^{l}$ with similar dimensions as $O^{l}$. The binary entries of the mask may follow a particular distribution (e.g., Bernoulli or Gaussian). Formally, Equation (1) is reformulated by:

(3) $$\begin{array}{c} {\tilde{O}}_{i}^{l}=M_{i}^{l}⊙O_{i}^{l} \end{array}$$

(4) $$\begin{array}{c} I_{i}^{l+1}=W_{i}^{l+1}{\tilde{O}}_{i}^{l}+b_{i}^{l+1} \end{array}$$

where ⊙ represents the element-wise multiplication. Although assuming random binary masks is straightforward, it may ignore crucial task-specific information. Therefore, in the following sections, alternative approaches are proposed to overcome this issue.

### 3.1. Approach 1: Mutual Information (MI) Dropout

Neural network layers map the input data *X* to a latent representation, *Z*, which has some desirable properties for the predefined network’s task. One of the crucial deep structures’ tasks is feature extraction, i.e., mapping the input to the latent space. Although each dimension of this latent space conveys some information to the output layer, usually, not all this information is necessary for a specific target of the NN’s task. In other words, some of the latent space’s dimensions are irrelevant to the target variable, and this irrelevant information could cause a disturbance in the prediction of the target variable [35]. Therefore, to prevent squandering computational resources and aggravation of the structure’s performance, the more relevant features must be considered in the latent space.

MI evaluates the relationship between two random variables, *X* and *Y*, from the entropy’s perspective. Entropy is a criterion for measuring the amount of uncertainty in a random variable. High entropy shows that each event has about the same likelihood of occurrence, while low entropy means different occurrence probabilities. Let $H_{P}\left[X\right]$ denote the entropy of a continuous variable *X* with instances *x* following probability density function (pdf) *P*. The differential entropy of *X* is computed by:

(5) $$H_{P}\left(X\right)=-\int_{-\infty }^{+\infty }P\left(x\right)log_{2}P\left(x\right)dx$$

Entropy is interpreted as the expected value of the negative logarithm of the probability distribution. The joint entropy of two variables *X* and *Y*, with a joint pdf $P\left(X,Y\right)$, is defined by:

(6) $$H_{P}\left(x,y\right)=-\int_{-\infty }^{+\infty }\int_{-\infty }^{+\infty }P\left(X,Y\right)log_{2}P\left(X,Y\right)\;dx\;dy$$

Based on the definition and considering (5) and (6) the MI between *X* and *Y* is calculated by:

(7) $$MI\left(X,Y\right)=\int_{-\infty }^{+\infty }\int_{-\infty }^{+\infty }P\left(X,Y\right)log_{2}\left(\frac{P\left(X,Y\right)}{P\left(X\right)\times P\left(Y\right)}\right)\;dx\;dy$$

(8) $$MI\left(X,Y\right)=H\left(X\right)+H\left(Y\right)-H\left(X,Y\right)$$

Based on (7), *I* is zero when *X* and *Y* are statistically independent, i.e., $P\left(X,Y\right)=P\left(X\right)\times P\left(Y\right)$.

Suppose a NN with *N* training samples, $\left\{\left(X_{1},Y_{1}\right),\left(X_{2},Y_{2}\right),\cdots ,\left(X_{N},Y_{N}\right)\right\}$, we define the activation matrix for all *M* hidden units of *l*th layer at each training step as:

(9) $$O^{l}=\left(\begin{array}{ccc} o_{11}^{l} & o_{12}^{l}\cdots & o_{1N}^{l}\\ \vdots & \ddots & \vdots \\ o_{M1}^{l} & o_{M2}^{l}\cdots & o_{MN}^{l} \end{array}\right)\in R^{M\times N}$$

where $o_{m,n}^{l}$, with $m\in \left\{1,2,\cdots ,M\right\}$ and $n\in \left\{1,2,\cdots ,N\right\}$ denotes the activation of *m*th neuron in layer *l* for the *n*th training sample. Therefore, each row of the matrix $O^{l}\in R^{M\times N}$ is the activation vector for a hidden neuron at each training step, we show this vector as $o_{m}^{l}$.

**Figure 1:**
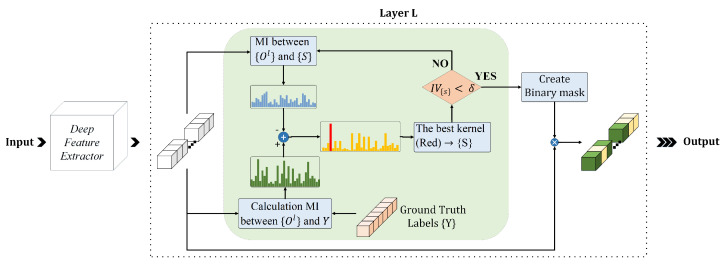
Applying MI dropout to the *l*th dense hidden layer.

The proposed approach, MI dropout, maintains a set of selected neurons, initialized as the empty set that are the most relevant set of kernels to the specified task. A two-fold function determines the task relevance of the latent units. In the first part, the MI between the hidden units’ activation and the target vector is calculated. The second part scores the hidden units by computing the MI between the remaining and the currently selected units. The higher relevancy between a neuron’s activation and target vectors, and the lower correlation with selected neurons, leads to a stronger chance for selection by MI dropout, i.e., the lower chance of removal. By combining these two parts in (10), we encourage the model to select units that are highly relevant to the target vector. These units have a low nonlinear correlation with each other. Given (7) and (8), our MI dropout merit function for neuron *i* at layer *l* is defined by:

(10) $$MF_{o_{i}^{l}}=MI\left(o_{i}^{l},Y\right)-\frac{\kappa }{\left|S\right|}\sum_{j=1}^{\left|S\right|}MI\left(o_{i}^{l},S_{j}\right)$$

where *Y* is the neuron’s activation vector, *S* denotes the set of selected latent neurons, |S| is the total number of selected neurons, and κ is a trade-off coefficient to define the trade-off between the quantity and quality of the information in the selection procedure. This coefficient is determined by the validation procedure. Please note that a higher merit function shows a lower chance of removal for a neuron. Figure 1 illustrates the pipeline of the MI dropout method. As shown in this figure, in each iteration, MI dropout evaluates all unselected neurons and the most valuable neuron (with the highest task-specific information computed in (10)) that is illustrated in red is added to the set of selected neurons.

The training flow of deep neural networks considering the MI dropout method is provided in Algorithm 1. The main goal is to select the neurons at layer *l* based on their performance on the whole set of training samples.
**Algorithm 1:** MI Dropout
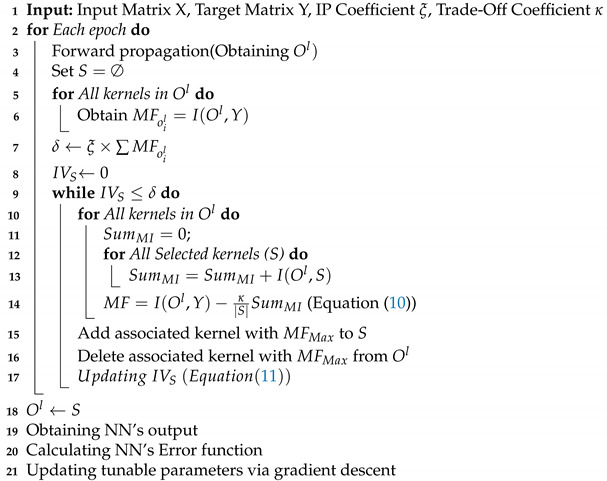


Initially, the set of selected kernels, *S*, is empty. Similar to other training algorithms, feed-forward propagation gives us the activation maps of all neurons in layer *l* for all training samples denoted by $O^{l}$. In the next step, we determine each neuron’s importance considering matrix $O^{l}$ in a while loop. The loop is terminated when the information volume (IV) for the subset *S* becomes more than a partition of all neurons’ IV. This part of information is determined by the information partitioning (IP) coefficient ξ. In this context, the information volume for each set is the sum of MI values between activation functions and output vectors. More formally, this value is obtained by:

(11) $$IV_{S}=\sum_{s}I\left(S,Y\right)$$

### 3.2. Approach 2: Determinantal Point Process (DPP) Dropout

In addition to Algorithm 1, we devise DPP dropout that works based on the Determinantal Point Process [13] to select the most diverse set of latent kernels. Promoting diversity leads to maintaining informative neurons while reducing the size of each latent layer.

A random point process P on a discrete base set $Y=\left\{1,\cdots ,N\right\}$ is a probability measure of all subsets of Y, denoted by $2^{Y}$. Let *S* be a positive semi-definite N×N matrix with columns and rows indexed by the elements of Y. P is a determinantal point process if, for a random subset Y drawn according to P, we have:

(12) $$\forall A\subseteq Y:P\left(A\subseteq Y\right)=det\left(S_{A}\right)$$

Here, *S* must be real positive semi-definite S⪯I; that is, all eigenvalues of *S* are in the range [0,1]. Since *S* denotes a probabilistic measure, all its principal minors must be non-negative. One can view *S* as a marginal kernel because it contains all the information needed to compute the probability of any subset *A* being selected in *Y*. $S_{A}$ represents the $\left|A\right|\times \left|A\right|$ submatrix of *S* indexed by the elements of *A*; that is, $S_{A}\equiv S_{\left[i,j\in A\right]}$ and by convention $det(S_{ø}=1)$.

Based on (12), the marginal probability for a subset with one and two items can be calculated by (13) and (14), respectively,

(13) $$\begin{array}{c} P\left(\{e_{i}\}\in Y\right)=S_{ii} \end{array}$$

(14) $$\begin{array}{c} P\left(\{e_{i},e_{j}\}\in Y\right)=S_{ii}S_{jj}-S_{ij}^{2} \end{array}$$

Equation (14) shows that the off-diagonal elements determine the negative correlations between pairs of elements; in other words, large values of $S_{ij}$ imply that elements *i* and *j* tend not to co-occur.

To model real data, we restrict DPPs by focusing on L-ensembles. An L-ensemble is a probability measure on $2^{Y}$ defined via a positive semi-definite matrix *L* indexed by the elements of Y such that:

(15) $$P_{L}\left(A\right)=\frac{det\left(L_{A}\right)}{det\left(I+L\right)}$$

where $L_{A}$ is a principal minor of matrix *L* regarding the elements in set *A*, and *I* is a N×N identity matrix. Using matrix decomposition, one can view $L_{ij}$ as a Gram matrix with elements $L_{ij}=q_{i}\phi_{i}^{T}\phi_{j}q_{j}$, where $q_{i}\in R^{+}$ represent the intrinsic quality of item *i*, and $s_{ij}=\phi_{i}^{T}\phi_{j}$ denotes the similarity between two items $q_{i}$ and $q_{j}$ where $\phi_{i}^{T}\phi_{j}\in \left[-1,1\right]$. Please note that L-ensemble is a DPP with marginal kernel *K* defined by:

(16) $$K=L{\left(L+I\right)}^{-1}=I-{\left(L+I\right)}^{-1}$$

Also, we compute the eigen decomposition of *L* using $\sum_{n=1}^{N}\lambda_{n}v_{n}v_{n}^{T}$; Hence, the marginal kernel *K* can be computed using a simple rescaling of eigenvalues:

(17) $$K=\sum_{n=1}^{N}\frac{\lambda_{n}}{\lambda_{n}+1}v_{n}v_{n}^{T}$$

Using (17) the number of objects in set *Y* is distributed as the number of successes in *N* Bernoulli trials, where trial *n* succeeds with probability $\frac{\lambda_{n}}{\lambda_{n}+1}$. More formally, the expected cardinality of *Y* is,

(18) $$E\left[\left|Y\right|\right]=\sum_{n=1}^{N}\frac{\lambda_{n}}{\lambda_{n}+1}=tr\left(K\right)$$

If we consider the neurons’ activation vectors in a layer of a deep structure as a set of discrete items, one can select the most diverse subset of kernels. From this point of view, DPP dropout promotes both quality and diversity of the kernels in the hidden layer of a deep NN to choose the best items that are dissimilar to each other. The selection of the hidden neurons using this approach, gives us a diverse (dissimilar) and task-relevant (high quality) subset of kernels.

Let us denote the total number of training samples by *N* while $N^{l}$ is the number of neurons in layer *l*, $o_{ij}^{l}$ is the output of *i*-th neuron in layer *l* on the *j*-th input sample, and $O_{i}^{l}=\left(o_{i1}^{l},o_{i2}^{l}\cdots ,o_{iN}^{l}\right)$ is the activation vector of the *i*-th neuron in layer *l* obtained by feeding the entire training dataset. To construct the matrix $L^{DPP}$ in (15), we make use of a Gaussian kernel which provides a good trade-off between the simplicity and precision of classification tasks:

(19) $$L_{ij}^{DPP}=exp\left(-\frac{\alpha }{N_{train}}{∥o_{i}^{l}-o_{j}^{l}∥}^{2}\right)+\epsilon I,\;1\leq i,j\leq K_{l}$$

where $K_{l}$ is the total number of neurons in layer *l*, and α determines the bandwidth of the Gaussian kernel. In this paper, we set α by evaluation of the results on a validation set. Please note that the matrix $L^{DPP}$ must be positive semi-definite. To ensure this, we add a diagonal matrix, ϵI to (19) with distribution rate ϵ=0.01.

Algorithm 2 is the pseudocode of the proposed DPP dropout. As with the MI dropout, we initially determine the output matrix of the *l*th layer, i.e., $O^{l}$. Then DPP dropout finds a diverse subset of these kernels, and finally, using error backpropagation, the NN parameters for the selected kernels are updated. The DPP dropout consists of two main phases. First, depending on the eigenvalues of the kernel matrix $L^{DPP}$, a random subset of eigenvectors *V* is selected. In the second phase, based on the selected eigenvectors, a sample set *S* is produced in a probabilistic fashion with probability P(i). At each iteration of the second loop, *S*’s cardinality increases by one; however, the dimension of V is reduced by one. In Algorithm 2, the vector $e_{i}$ is a binary vector that is all zeros except for a one at the *i*th position.
**Algorithm 2:** DPP Dropout
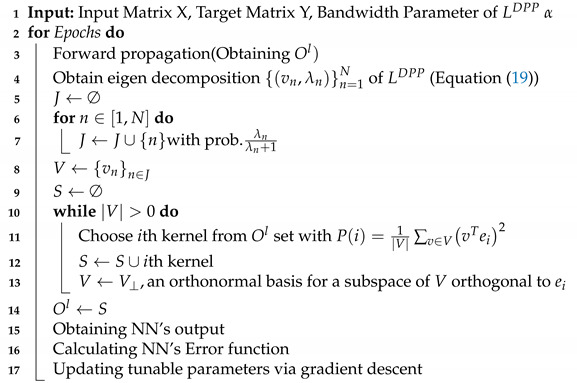


### 3.3. Approach 3: Determinantal Point Process Mutual Information (DPPMI) Dropout

As shown in Section 3.1 and Section 3.2, one can select the most informative set of hidden neurons, as well as the most diverse subset of kernels to cover the latent space with lower number of dimensions. Subsequently, integrating MI approach into DPP sampling gives us the most informative and diverse subset.

**Figure 2:**
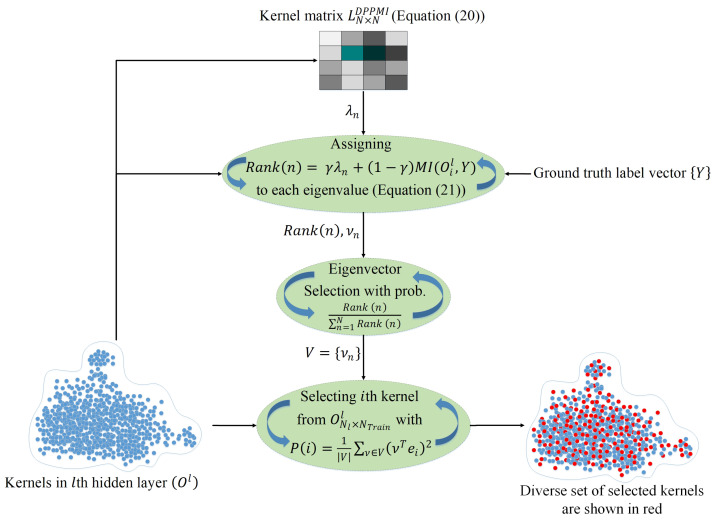
DPPMI dropout. Each point illustrates an extracted hidden kernel in the training set.

Figure 2 illustrates the proposed DPPMI dropout. As depicted in this figure, the goal is to find a diverse subset of neurons in the *l*th layer in which negligible information is shared among neurons due to their high diversity. In addition, the selected kernels are highly related to the output target as a result of leveraging the MI metric. In this approach, we define a new kernel matrix *L*:

(20) $$L_{ij}^{DPPMI}=exp\left(-\left(\frac{\alpha }{N_{train}}{∥o_{i}^{l}-o_{j}^{l}∥}^{2}+\frac{\beta }{MI\left(O_{i}^{l},O_{j}^{l}\right)+\epsilon }\right)\right)$$

where α and β hyper-parameters determined by a validation set. Considering the fact that $\forall i,j\;i\neq j\;MI(O_{i}^{l},O_{j}^{l})\geq 0$ and $\forall i,\;MI(O_{i}^{l},O_{i}^{l})\neq 0$, the defined kernel function in (20) is certainly positive semi-definite; therefore, we do not need to add a diagonal matrix to $L^{DPPMI}$. However, a small value ϵ>0 must be added to $MI\left(O_{i}^{l},O_{j}^{l}\right)$ to avoid increasing $\frac{\beta }{MI\left(O_{i}^{l},O_{j}^{l}\right)+\epsilon }$. In contrast to Algorithm 2 that selects the kernels based on the eigenvalues of matrix *L*, the proposed DPPMI dropout selects kernels using a rank criterion that considers both eigenvalues and the MI among kernels and the output vector. The rank is computed by:

(21) $$Rank\left(n\right)=\gamma \lambda_{n}+\left(1-\gamma \right)MI\left(O_{i}^{l},Y\right)$$

where $\lambda_{n}$ is the *n*th eigenvalue of matrix $L^{DPPMI}$ and γ is a hyper-parameter that reflects the contribution rate for each term in (21).

Algorithm 3 shows the details of the DPPMI dropout approach. The proposed DPPMI dropout selects a diverse subset of kernels that are most relevant to the underlying classification task. Our proposed approach constructs $L^{DPPMI}$ considering both Euclidean similarity and MI of latent kernels, and selects the kernels based on the eigenvalues of $L^{DPPMI}$ as well as the MI between each kernel with ground truth label vector.
**Algorithm 3:** DPPMI Dropout
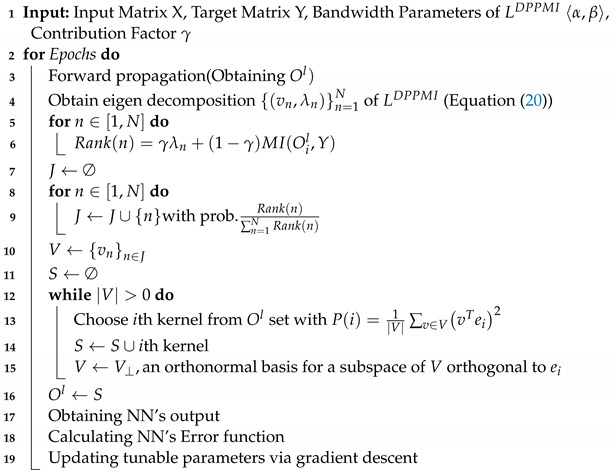


### 3.4. Inference Procedure

As shown in Algorithms 1–3, we merely apply dropout during the training stage of neural network. Similar to [10], here, we compensate the effect of dropout by using a single neural network at test time without dropout. The weights of this neural network are scaled-down versions of the trained weights. Although Standard dropout assumes a constant downscaling rate for all neurons, our approach defines a unique rate corresponding to each neuron. We determine the rate of each neuron by counting the number of times it is retained during the training phase. Formally, the downscaling rate for the *i*th neuron at layer *l* is obtained by:

(22) $$p_{n_{i}^{l}}=\frac{N_{r_{i}}}{E}$$

where $N_{r_{i}}$ is the number of times neuron *i* at layer *l* is retained while *E* is the number of total training epochs.

## 4. Experimental Results and Discussion

In this section, we evaluate the performance of our proposed methodologies: DPP dropout, MI dropout, and DPPMI dropout detailed in Section 3.1, Section 3.2 and Section 3.3 on widely used datasets for image classification tasks. Moreover, the proposed approaches are compared with several mainstream dropout techniques in terms of classification accuracy.

### 4.1. Datasets

**Figure 3:**
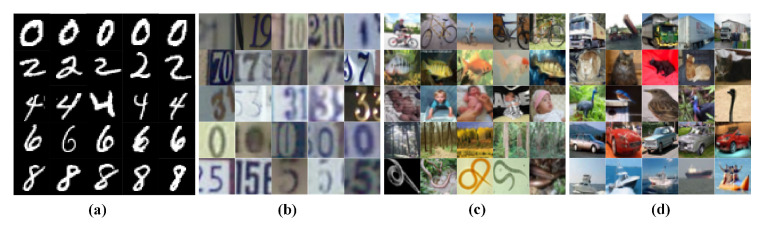
Example images from (**a**) MNIST, (**b**) SVHN, (**c**) CIFAR10, (**d**) CIFAR100 datasets. Only random samples of five classes are shown, and each row corresponds to a different category.

We evaluate the models’ performance on large-scale image datasets: MNIST [36], SVHN [37], CIFAR10 and CIFAR100 [38]. The MNIST consists of 70,000 grey-scale images of handwritten digits (from 0 to 9), with resolution 28×28. We used 60,000 samples of images for training and the remaining testing. The SVHN includes real-world 32×32 RGB images of digits. The training and testing phases use 73,257 and 26,023 digits, respectively. Two well-known object recognition datasets, CIFAR10 and CIFAR100, consist of 32×32 tiny RGB images in 10 and 100 different categories. For both datasets 50,000 training and 10,000 testing images are considered. Figure 3 shows several samples of the underlying datasets.

### 4.2. Implementation Details

For the MNIST and SVHN dataset, we construct a model consisting of two hidden layers with 750 and 350 hidden units and rectified linear unit (ReLU) activation functions. For MNIST the model includes 784 and for the greyscale SVHN the model consists of 1024 input and 10 output nodes with ReLU and SoftMax activation functions, respectively. For more challenging datasets, CFAR10, and CIFAR100, the pre-trained VGG16 [39] and ResNet50 [40] are employed for feature extraction. The extracted features from VGG16 and ResNet50 are fed to two subsequent fully connected layers with 700 and 350 hidden units. We initialize the tunable parameters using the Xavier method [41]. To train our models, we make use of a cross entropy loss function using a 200-epoch stochastic mini-batch gradient descent method with a batch size of 128, a fixed learning rate of 0.001, a momentum value of 0.9, and a weight decay equal to $5\times 10^{-4}$.

In all the implemented models, we applied the dropout layers after the first dense layer. For MI calculation among kernels, we exploit the open-source non-parametric entropy estimation toolbox [42] and to implement the DPP method, we exploit the toolbox from [43].

**Figure 4:**
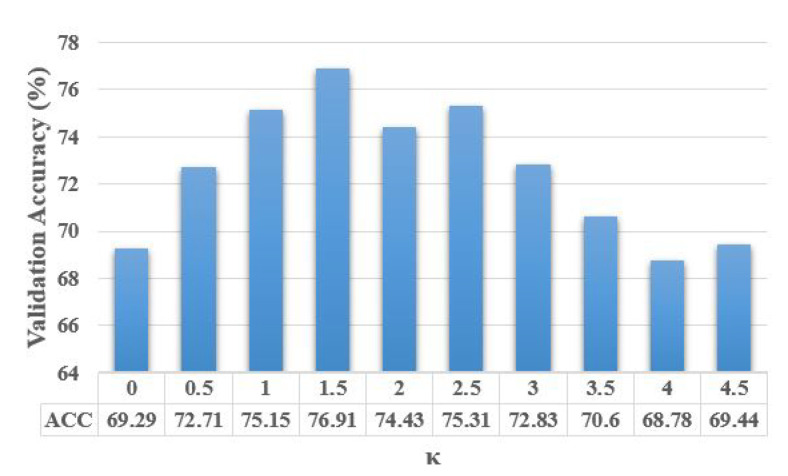
Validation accuracy of MI dropout model with different configurations of defined hyper-parameter κ in (10) on the CIFAR10 dataset.

**Figure 5:**
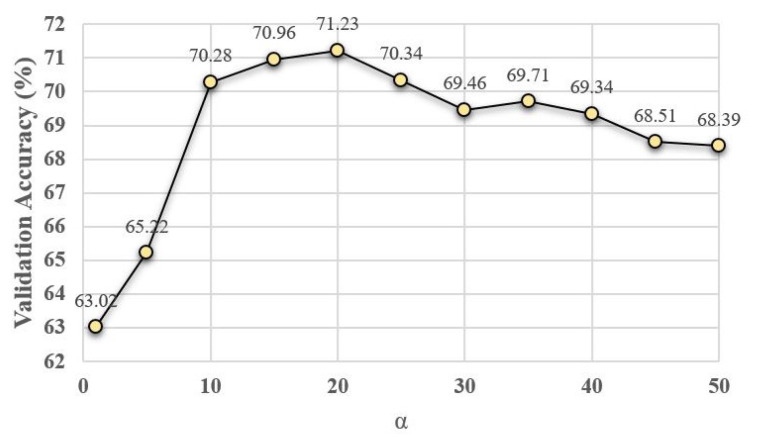
Validation accuracy of DPP dropout model with different configurations of defined hyper-parameter α in (19) on the CIFAR10 dataset.

**Figure 6:**
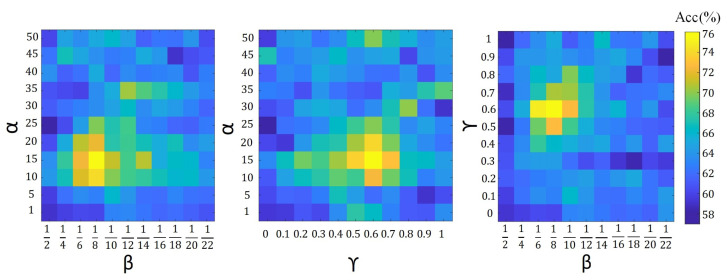
Validation accuracy of DPPMI dropout with different configurations of defined hyper-parameters in (20) and (21).

To analyze the contribution of our hyper-parameters, i.e., κ in (10), α in (19), and $\langle \alpha ,\beta ,\gamma \rangle$ in (20) and (21), on the accuracy of proposed algorithms, we show the validation accuracy of their different configurations. For each setting of our search space, the model is trained on the training set and evaluated on the validation set. The configuration with the highest validation accuracy is chosen as the optimal model and further evaluated on the testing set. The validation search space for different parameter configurations is defined by $\kappa \in \left\{0,0.5,1,1.5,2,2.5,3,3.5,4,4.5\right\}$ $\alpha \in \left\{1,5,10,15,20,25,30,35,40,45\right\}$, $\beta =\frac{1}{2i}$ where i∈[1,11] and 0≤γ≤1. Figure 4, Figure 5 and Figure 6 show the average validation accuracy for all considered configurations of hyper-parameters in (10), (19), (20) and (21) on the CIFAR10 dataset. As shown in Figure 4, Figure 5, and Figure 6, the optimal configurations of hyper-parameters for MI dropout, DPP dropout and DPPMI dropout are κ=1.5, α=20, and $\langle \alpha ,\beta ,\gamma \rangle =\langle 15,\frac{1}{8},0.6\rangle$, respectively.

To verify the effectiveness of our proposed algorithms, we compare our approaches with recent dropout techniques, including the Automatic dropout [17], Controlled dropout [22], DropMI dropout [24], Guided dropout [15], Concrete dropout [23], and Targeted dropout [18], as well as the Standard dropout [10]. All the experiments are carried out using GPU-based Tensorflow [44] on Python 3. The simulations are processed in a system with a 10-core CPU with Intel core-i7 Processors, an NVidia Quadro RTX 6000 GPU, and a 256-GB RAM.

### 4.3. Numerical Results

Table 1 compares the proposed approaches’ classification performance with the referred state-of-the-art algorithms on four benchmark datasets. In line with the observations in [40,45], our results on CIFAR10 and CIFAR100 datasets verify the superiority of the ResNet50 over the VGG16 feature extractor backbones. Also, the obtained results verify the capability of dropout methods in overfitting mitigation [10]. For example, by comparing the results of Standard dropout and No dropout, one can observe that with VGG16 feature extractor on CIFAR10 and CIFAR100 datasets, Standard dropout increases the test classification accuracy 5.15% and 3.97%, respectively. From comparing the obtained result between Standard dropout and Automatic dropout, we found that Automatic dropout works slightly better than the Standard dropout; for example, on the SVHN dataset, the Automatic dropout enhanced by 1.11%. The results show the same behavior in slightly better performance of Automatic dropout over Standard dropout for CIFAR10 and CIFAR100 (e.g., with ResNet50 backbone 0.79% and 0.33% improvements, respectively). The reason for this observation is the existing similarity between Automatic dropout and Standard dropout. Like Standard dropout, in Automatic dropout, the *p* coefficient is considered randomly from a Gaussian probability distribution, N, but each cluster of activation functions has its own *p*. Similar to [22], the obtained results verify that Controlled dropout gained a little improvement compared to Standard dropout (e.g., with VGG16 feature extractor 0.71% and 0.62% on CIFAR10 and CIFAR100, respectively) in the neural network’s performance, despite having lower memory usage.

According to Table 1, Guided dropout achieves higher performance across all datasets than Standard dropout, Automatic dropout, and Controlled dropout. For example, one can observe that with the VGG16 backbone, Guided dropout’s test accuracy on CIFAR10 and CIFAR100 are 66.11% and 43.09%, respectively, while these values for Controlled dropout are 64.73% and 40.87. This enhancement in the classification accuracy is because, in contrast to previous methodologies that generally determine the *p* parameter randomly, Guided dropout seeks to optimize the *p* parameter; hence, picking the neurons in a more informative manner.

Concrete dropout achieves higher performance than the Standard dropout, Automatic dropout, and Controlled dropout. For instance, compared to the Controlled dropout on the SVHN and CIFAR100 datasets with ReseNet feature extractor, Concrete dropout obtains 2.5% and 3.34% improvements, respectively. This is because Concrete dropout finds an optimized rate for *p* and the model’s tunable weights with respect to the defined variational interpretation-based objective function. Moreover, one can observe the small improvement of the results by Concrete dropout over Guided dropout. With ResNet50 feature extractor on CIFAR10 and CIFAR100, the Concrete dropout test accuracies are 69.01% and 46.51%, while these values for Guided dropout are 68.32% and 45.87%, respectively. The reason for this improvement in the results is that Concrete dropout considers the unique and more advanced objective function; however, Guided dropout simply finds the neurons’ contribution based on the model’s error.

In contrast to previous approaches that deal with all NN’s neurons, by applying the dropout on the portion of neurons with low-importance connections, i.e., neurons with small $∥W∥$, Targeted dropout with 98.33% and 69.09% test accuracies achieves the best results among referred baselines on MNIST and SVHN datasets, respectively. However, for more complex datasets, CIFAR10 and CIFAR100, DropMI with 71.21% and 47.23% on ResNet50 backbone, gains the highest accuracy in comparison with other dropout algorithms. Generally speaking, the obtained results show a similar performance of Targeted dropout and DropMI dropout. The reason for the superiority of DropMI dropout over Targeted dropout on CIFAR10 and CIFAR100 is that Targeted dropout does not consider conveying information from latent kernels to the output, while DropMI determines the important kernels by computing the MI between kernels and the target vector. This information volume plays a critical role in solving the more challenging tasks such as colored image recognition, i.e., CIFAR10 and CIFAR100.

As shown in Table 1, the proposed DPP dropout method in Section 3.2 that selects the diverse set of latent kernels has a better performance compared to No dropout. For example, notice the 5.56% and 8.05% improvement on the test accuracy of No dropout by DPP dropout on SVHN and CIFAR10 (with VGG16 backbone) datasets, respectively. This superiority concludes that the concept of diversity in selecting kernels must be considered in the deep latent space. Moreover, DPP dropout shows a higher test accuracy in comparison to the Standard dropout with 2.15% and 2.75% improvements on CIFAR10 and CIFAR100 respectively using ResNet50 feature extractor. The reason for this observation sheds light on the difference between random sampling and DPP sampling. However, since the DPP dropout keeps the kernels merely based on the concept of diversity in the kernel space and does not consider the importance of the kernels to the underlying task, it shows a lower test accuracy than other baselines across all datasets.

As reported in Table 1, the proposed MI dropout with VGG16 achieves the test classification accuracy of 70.91% and 47.11% on CIFAR10 and CIFAR100, respectively. The proposed MI dropout outperforms the best mentioned dropout method on CIFAR10 and CIFAR100 (with VGG16 backbone), i.e., DropMI dropout, by 1.58% and 2.06%. This is because, in contrast to DropMI, which merely selects the kernels that include high MI with the target vector, the proposed MI dropout minimizes the shared information between selected kernels, and chooses highly relevant task-specific kernels. This superiority concludes that some kernels in the latent space of deep NNs convey similar information to their subsequent layer. Hence, choosing the set of kernels with high MI with target vector and with low MI between each other can help mitigate overfitting, i.e., increasing the test accuracy.

Furthermore, integrating MI into DPP dropout by defining a new kernel matrix and selection probability rank helps DPPMI dropout to achieve a higher test accuracy among DPP dropout, MI dropout, as well as the different referred baselines. Based on Table 1, DPPMI dropout outperforms the best algorithms among baselines, i.e., Targeted dropout on digit recognition and DropMI on colored object recognition. The proposed DPPMI improves the accuracy of Targeted dropout by 0.45% and 2.24% on MNIST and SVHN datasets; also, it outperforms DropMI with ResNet50 backbone by 3.35% and 3.13% on CIFAR10 and CIFAR100. This observation is because the DPPMI dropout considers the diverse and most relevant kernels set to the underlying task. By comparing the obtained results among DPPMI dropout, DPP dropout, and MI dropout algorithms, one can notice that DPPMI dropout shows better performance compared to MI dropout. For instance, DPPMI dropout method with VGG16 feature extractor yields 1.21% and 0.93% improvements compared to MI dropout on CIFAR10 and CIFAR100, respectively, while in the same comparison between DPPMI dropout and DPP dropout, one can observe 5.20% and 5.03% improvement in test accuracy of DPP dropout by DPPMI dropout. This observation concludes that the concept of quality and quantity of information is more critical than selected kernels’ diversity.

### 4.4. DPPMI Dropout as Model Compression

**Figure 7:**
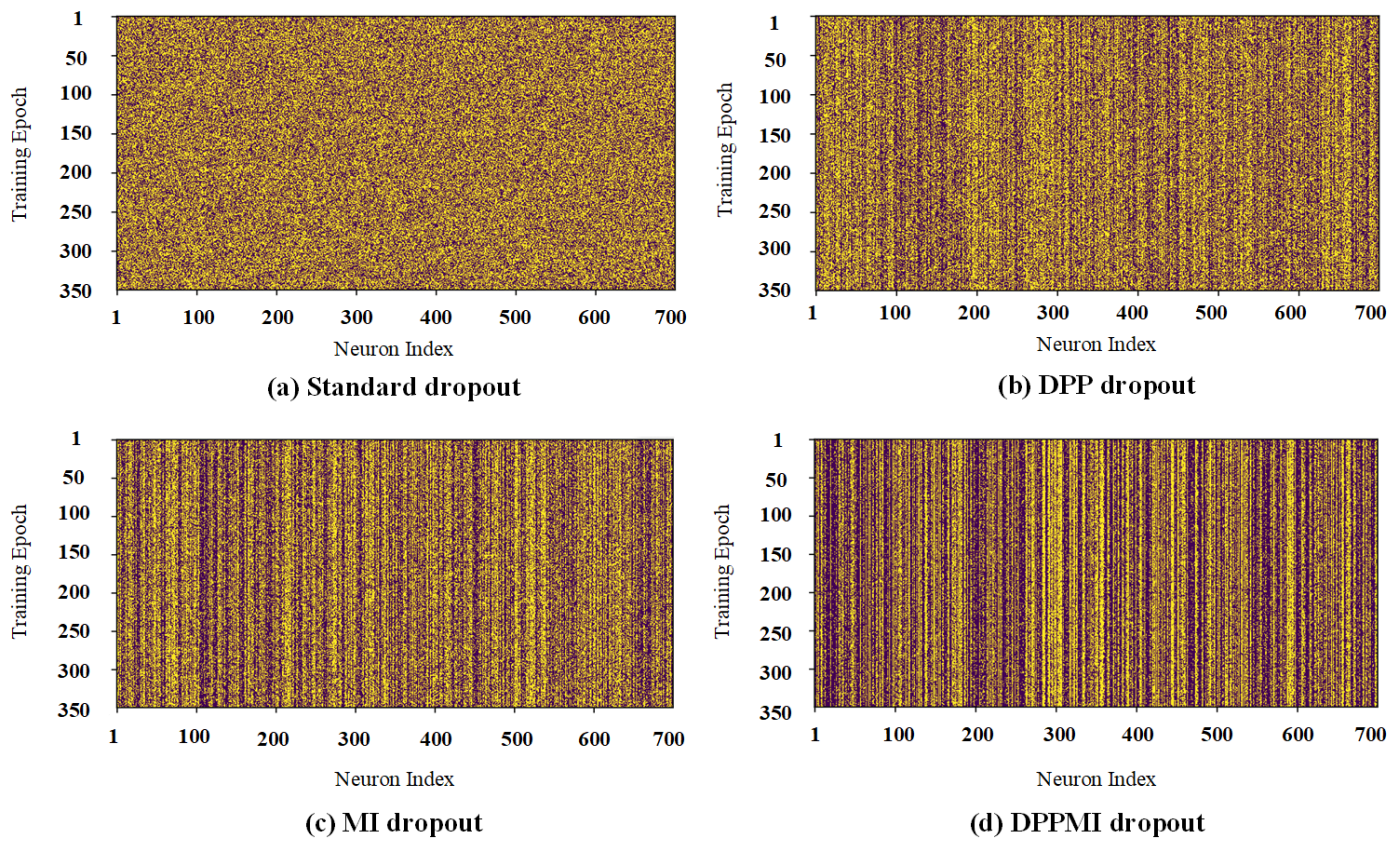
Activation maps of the first dense layer in (**a**) Standard dropout; (**b**) DPP dropout; (**c**) MI dropout; (**d**) DPPMI dropout. Yellow and purple pixels depict active and inactive neurons, respectively.

Our proposed approaches aim to avoid randomness in dropout selections and retain the neurons based on their performance in the training phase. Figure 7 visualizes the neuron activations in the first dense hidden layer of a network trained on the CIFAR10 dataset. Each row of the visualized matrices in Figure 7 represents the dense layer’s activation map for the entire training phase. The dark pixels show the inactive neurons, and the bright ones show the active neurons in the model. In this figure, we notice the difference in activated neurons in Standard dropout and DPP dropout. As expected, the selection in the Standard dropout is random; thus, this method gives a noisy activation map. However, DPP dropout selects the set of neurons based on the diversity criterion. Therefore, in this method, some neurons gained a higher probability rate compared to other neurons.

From the comparison corresponding to the activation maps of DPP dropout and MI dropout, one can see that MI dropout is choosing a lower number of neurons in each training epoch. At initial steps, MI dropout determines and retains the more informative neurons and updates their corresponding weights. Since MI dropout only trains the corresponding weights of the informative kernels, the probability of selecting these kernels will increase at each training step. Obviously, some neurons contribute (are active) in the underlying task for almost every training epoch; thus, they earn a lower probability of being removed. However, some neurons have less contribution in the model; hence, they gain less probability. Please note that compared to other activation maps in Figure 7, in the DPPMI dropout’s activation map, there are more activated units because it selects neurons more strictly at each training epoch.

**Figure 8:**
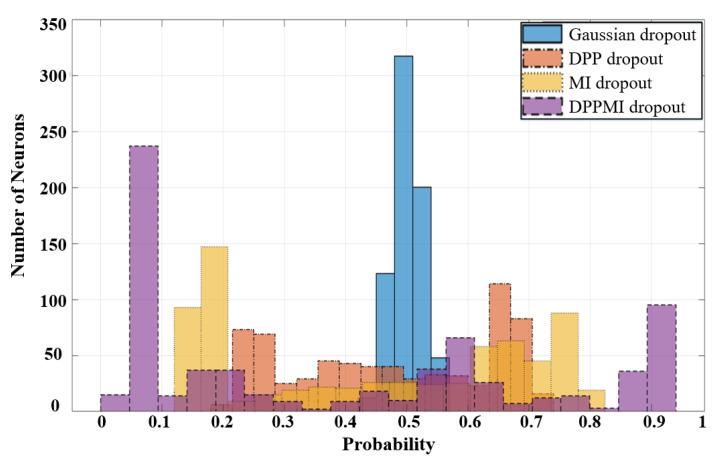
Assigned probabilities to neurons on different approaches.

Based on the obtained masks in Figure 7 and using (19), we calculate the dropout probability for every single neuron in the model. Figure 8 compares the histogram of the neurons’ probability for standard Gaussian dropout and the proposed approaches. One can observe that DPPMI dropout determines the most active neurons, i.e., the neurons that gain p>0.9, as well as the least active neurons, i.e., the neurons that earn p<0.1, in the highest and lowest probability regions of the histograms in Figure 8, respectively. Considering this rate of contribution gives us a better sight for pruning the model. More concretely, in the case of model compression, we can prune the model by removing the set of neurons with a lower p<η; however, it should be emphasized that determining the threshold rate, η, depends on the dataset and the underlying task.

### 4.5. Discussion on Overfitting Prevention

To investigate the capability of overfitting prevention for the proposed dropout methods, similarly to [45], we conduct two experiments. Deep neural networks encounter the overfitting issue due to having a large parameter space and a relatively low number of training examples. As mentioned in Section 4.2, we extract the features using the VGG16 [39] baseline. The deep visual features are fed to dense layers. In the first experiments, we increase the model’s parameters by adding some hidden fully connected layers. Thus, in each step of this experiment, we consider 2, 4, and 8 dense layers and each layer consists of 800 neurons.

Table 2 shows the numerical results of these experiments on the CIFAR10 dataset. Comparing this table’s columns, we notice that with the increase of model’s depth, the performance generally drops for all algorithms. When we compare the first and the third columns of the model on No dropout row, we see that the performance drops by 16.14%. However, this value for other baselines such as Guided dropout and Controlled dropout is less than 11%. This shows the importance of dropout methods to help avoid overfitting while providing high generalization capacity in very deep neural architectures. A similar analysis for the proposed DPPMI dropout and the best baseline on CIFAR10 dataset, DropMI dropout, shows that the performance declines by 6.96% and 8.30%, respectively.

In the second experiment, during the training phase, we decrease the number of training samples uniformly for each class of the CIFAR10 dataset, while the number of hidden layers is fixed as explained in Section 4.2. In this experiment, we defined a coefficient ϵ that represents the relative number of training samples compared to the original size of the training data. For example, when ϵ=0.6, for each class of the CIFAR10, we randomly choose 50,000 × 0.6 = 30,000 samples for the model’s training while the number of testing samples remains the same as the original dataset.

Table 3 shows the results of this experiment. According to this table, with the decrease in ϵ, the test classification accuracy of the underlying model with different dropout approaches drops dramatically as expected. The gap between ϵ=0.6 and ϵ=0.2 columns for No dropout and Standard dropout is 19.06% and 12.97%, respectively. Hence, similar to the first experiment’s results, the obtained results in the second experiment reveal the role of dropout layer in model’s generalization enhancement. As shown in the table, the DPPMI dropout achieves the best performance on the three cases with different ϵs. In the table, we compare the results of the best baseline, i.e., DropMI dropout, and the proposed DPPMI dropout. The gap between the results of DropMI dropout for ϵ=0.6 and ϵ=0.2 is 10.02%, while the same metric for DPPMI dropout is 8.81%. Moreover, a similar analysis shows that the existing gap for MI dropout is 9.82%; therefore, the DPPMI dropout makes almost 1% improvement in the model’s generalization compared to proposed MI dropout. As a result, the designed experiments strongly conclude that with the increment of model parameters and the shrinkage of the training dataset, the proposed DPPMI dropout approach outperforms all recent benchmarks.

### 4.6. Empirical Time Complexity Analysis

**Figure 9:**
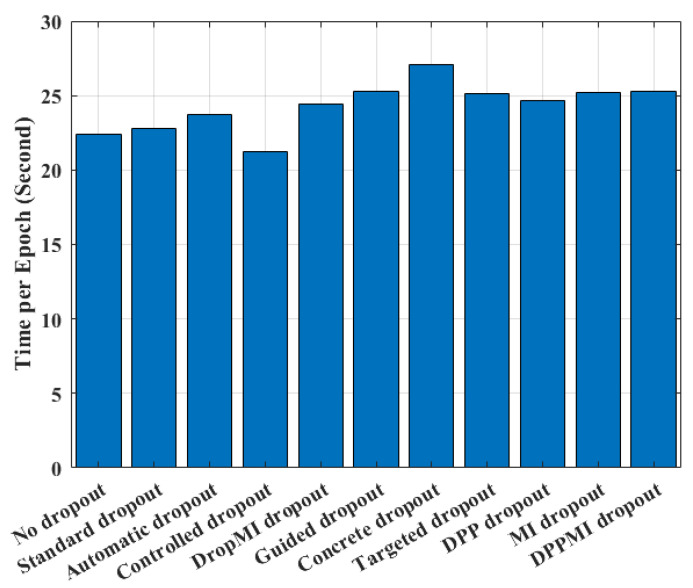
Comparison of the elapsed time per epoch for different methods.

Figure 9 illustrates the average running time per training epoch for the presented DPP dropout, MI dropout, as well as the DPPMI dropout with the recent benchmarks. The reported times are recorded for the CIFAR100 dataset using our computer architecture described in Section 4.2. As shown in this figure, the Standard dropout and Controlled dropout increase the epoch time of No dropout approach by 0.37 and 1.3 s. Also, DropMI dropout increases the training time by 1.68 s in comparison with Standard dropout, since DropMI calculates the MI value of all kernels with target vector in each training epoch. The Concrete and Guided dropout algorithms show 4.33 and 2.54 s higher running time than Standard dropout due to solving an optimization problem for adjusting suitable *p* parameter in dropout. As shown in this figure, the presented algorithms have a reliable empirical complexity with a small increase compared to the Automatic dropout and Targeted dropout, and a slight decline compared with recent state-of-the-art benchmarks such as Guided dropout and Concrete dropout. Our observation clearly justifies the use of our method in classification applications as it provides a significant accuracy improvement with a reasonable computational burden.

### 4.7. Comparison with Batch Normalization

Batch Normalization (BN) [8] is another regularization method that aims to change the distributions of internal neurons of a deep model in training phase to reduce internal covariate shift. Whitening (normalizing to obtain zero mean and unit variance [46]) of the inputs of each layer is the fundamental idea of BN to achieve the fixed distributions of inputs that would diminish the ill effects of the internal covariate shift and accelerate convergence of deep neural architectures [8]. Numerous recent studies have shown that combining dropout algorithms with BN needs caution since it leads to inconsistencies in internal variance of units which causes high classification error rates during both train and test stages [47,48,49].

In this section, we investigate the relationship between the proposed dropout algorithms and the BN method on training deep learning models. The experiment is carried out on SVHN and CIFAR10 datasets, and the classification baselines for these two datasets is considered similar to the explained baseline in Section 4.2. In this experiment, we exploit ResNet50 to extract the features from CIFAR10, also the regularization methods are only applied on the dense layers in the baseline. To see the effect of variance shift between training and testing datasets, we consider two sets of configurations between the dropout and BN layers: (**A**) dropout After BN layer (**B**) dropout Before BN layer. All the experimental settings, i.e., number of epochs, learning rate, weight initialization, batch size, etc. are considered similar to the settings in Section 4.2.

Table 4 compares the train/test accuracies obtained after training models with the incorporation of BN into the proposed dropout algorithms as well as the Standard dropout. By comparing the obtained results of Table 1 and Table 4, one can observe that the BN and Standard dropout show similar performance in terms of train and test accuracies in the training of the deep NNs. As shown in Table 4, the (**A**) configuration shows significantly better results compare to (**B**), especially in the test phase. This concludes that combining these two methods, i.e., dropout and BN, does not necessarily lead to the better generalization; hence, the results verify that the covariate shift happens when there exists a BN layer after the dropout layer [47,50]. The whitening effect of the BN layer on the latent kernels would lead to a more uniform latent space, thus, selecting the diverse group of features would be easier for our DPP procedure. As a result, the accuracies shown in Table 4 have consistent improvements when adding the BN layer before the dropout.

## 5. Conclusions

This paper presents a novel dropout strategy that semantically selects the neurons to be dropped in the latent layer of deep neural architecture. First, we assess the performance of each individual neuron in a training step, and afterward, we construct a binary mask that eliminates irrelevant neurons based on the concepts of quality and quantity as well as the diversity of the selected kernels. By integrating the mutual information and determinantal point process sampling, the proposed DPPMI dropout algorithm can activate hidden neurons that are highly correlated with the underlying neural network’s classification task. Numerical results on various classification benchmark datasets such as MNIST, SVHN, CIFAR10, and CIFAR100 verify that the proposed method can boost the generalization of the deep neural network and improve the classification accuracy of the model. Compared to state-of-the-art dropout methods, such as Guided dropout and Targeted dropout, the proposed work enhances the test classification accuracy by 4.95% and 3.43%, while maintaining a similar time complexity in the CIFAR100 dataset. Future works include designing a global search methodology for the automatic determination of hyper-parameters in the algorithms. Furthermore, the proposed work will be extended to improve convolutional neural architectures and recurrent structures.

## Figures and Tables

**Table 1:** Classification accuracies (%) on the digit recognition (MNIST and SVHN) and object recognition (CIFAR10 and CIFAR100) tasks. Please note that the best test results are marked in **bold** fonts and the best results among the underlying baselines are marked by underline, respectively.

Model	MNIST		SVHN		CIFAR10		CIFAR100	
	784-750-350-10		1024-750-350-10		VGG16/ResNet50		VGG16/ResNet50	
	Train	Test	Train	Test	Train	Test	Train	Test
No dropout	99.21	97.03	87.46	60.59	90.47/91.52	58.87/60.11	77.10/79.36	36.28/38.92
Standard dropout (p=0.5) [10]	99.14	98.25	80.66	64.10	86.59/88.63	64.02/66.10	62.65/64.73	40.25/42.36
Automatic dropout [17]	99.12	98.26	79.88	65.21	86.12/87.31	63.79/66.89	62.40/64.19	40.43/42.69
Controlled dropout [22]	98.98	98.28	76.61	64.91	87.26/88.88	64.73/67.04	62.12/63.36	40.87/43.17
DropMI dropout [24]	99.09	98.27	79.12	68.81	85.46/87.29	69.33/71.21	61.44/63.69	45.05/47.23
Guided dropout [15]	99.01	98.29	79.41	65.24	85.01/87.10	66.11/68.32	63.78/65.92	43.09/45.87
Concrete dropout [23]	99.11	98.31	79.72	67.41	85.52/87.74	66.91/69.01	61.13/63.15	44.17/46.51
Targeted dropout [18]	99.17	98.33	80.01	69.09	86.21/88.44	68.95/70.32	61.84/63.36	44.61/47.11
DPP dropout	99.14	98.24	80.43	66.15	85.11/87.26	66.92/68.25	62.91/64.82	43.01/44.11
MI dropout	99.01	98.72	78.47	70.11	85.42/86.61	70.91/73.02	61.23/63.40	47.11/49.52
DPPMI dropout	99.04	**98.78**	79.29	**71.33**	86.23/88.64	**72.12**/**74.56**	62.43/64.78	**48.04**/**50.36**

**Table 2:** Test accuracies of dense neural networks on CIFAR10 benchmark. The models’ structures are illustrated by (number of hidden layers × number of hidden units). The best test results are marked in **bold** fonts.

Model	Dense	Dense	Dense
	(2 × 800)	(4 × 800)	(8 × 800)
No Dropout	57.06%	51.94%	40.92%
Standard dropout (p=0.5) [10]	62.61%	58.99%	51.68%
Automatic dropout [17]	62.77%	59.04%	51.98%
Controlled dropout [22]	63.04%	59.39%	52.47%
DropMI dropout [24]	68.96%	65.52%	60.66%
Guided dropout [15]	65.94%	62.74%	56.55%
Concrete dropout [23]	65.10%	61.78%	55.65%
Targeted dropout [18]	68.06%	65.13%	59.18%
DPP dropout	65.85%	62.32%	56.21%
MI dropout	70.13%	67.81%	62.71%
DPPMI dropout	**71.49%**	**69.43%**	**64.53%**

**Table 3:** The results of test accuracies regarding different training set sizes on CIFAR10 benchmark. The parameter ϵ determines the portion of training sample number per class from original dataset. The best test results are marked in **bold** fonts.

Model	ϵ=0.6	ϵ=0.4	ϵ=0.2
No Dropout	56.09%	51.08%	40.03%
Standard dropout (p=0.5) [10]	62.07%	58.21%	49.10%
Automatic dropout [17]	61.92%	58.46%	49.38%
Controlled dropout [22]	62.88%	59.35%	50.39%
DropMI dropout [24]	68.12%	65.24%	58.10%
Guided dropout [15]	65.34%	62.09%	54.28%
Concrete dropout [23]	65.50%	62.19%	54.10%
Targeted dropout [18]	67.52%	64.51%	57.41%
DPP dropout	65.21%	61.52%	52.51%
MI dropout	69.81%	66.91%	59.99%
DPPMI dropout	**71.21%**	**69.07%**	**62.40%**

**Table 4:** Comparisons of train/test accuracies for various benchmarks using batch normalization and dropout algorithms. (**A**) and (**B**) denote dropout layer After and Before BN layer, respectively.

Model	SVHN		CIFAR10	
	Train	Test	Train	Test
No dropout + BN	79.22%	63.93%	88.24%	65.16%
Standard dropout + BN (**A**)	83.29%	67.36%	90.61%	69.07%
DPP dropout + BN (**A**)	83.09%	67.91%	89.93%	70.11%
MI dropout + BN (**A**)	80.11%	72.32%	89.21%	75.15%
DPPMI dropout + BN (**A**)	81.46%	74.20%	90.32%	77.19%
Standard dropout + BN (**B**)	79.10%	62.22%	86.84%	61.36%
DPP dropout + BN (**B**)	80.72%	63.09%	85.97%	64.26%
MI dropout + BN (**B**)	77.36%	67.75%	86.98%	69.36%
DPPMI dropout + BN (**B**)	79.29%	68.66%	87.79%	71.42%
